# Associations between multimorbidity, all-cause mortality and glycaemia in people with type 2 diabetes: A systematic review

**DOI:** 10.1371/journal.pone.0209585

**Published:** 2018-12-26

**Authors:** Jason I. Chiang, Bhautesh Dinesh Jani, Frances S. Mair, Barbara I. Nicholl, John Furler, David O’Neal, Alicia Jenkins, Patrick Condron, Jo-Anne Manski-Nankervis

**Affiliations:** 1 Department of General Practice, University of Melbourne, Melbourne, Australia; 2 General Practice and Primary Care, Institute of Health and Wellbeing, University of Glasgow, Glasgow, United Kingdom; 3 Department of Medicine, St Vincent’s Hospital, University of Melbourne, Melbourne, Australia; 4 NHMRC Clinical Trials Centre, University of Sydney, Sydney, Australia; 5 Brownless Biomedical Library, University of Melbourne, Melbourne, Australia; Chinese Academy of Medical Sciences and Peking Union Medical College, CHINA

## Abstract

**Introduction:**

Type 2 diabetes (T2D) is a major health priority worldwide and the majority of people with diabetes live with multimorbidity (MM) (the co-occurrence of ≥2 chronic conditions). The aim of this systematic review was to explore the association between MM and all-cause mortality and glycaemic outcomes in people with T2D.

**Methods:**

The search strategy centred on: T2D, MM, comorbidity, mortality and glycaemia. Databases searched: MEDLINE, EMBASE, CINAHL Complete, The Cochrane Library, and SCOPUS. Restrictions included: English language, quantitative empirical studies. Two reviewers independently carried out: abstract and full text screening, data extraction, and quality appraisal. Disagreements adjudicated by a third reviewer.

**Results:**

Of the 4882 papers identified; 41 met inclusion criteria. The outcome was all-cause mortality in 16 studies, glycaemia in 24 studies and both outcomes in one study. There were 28 longitudinal cohort studies and 13 cross-sectional studies, with the number of participants ranging from 96–892,223. Included studies were conducted in high or upper-middle-income countries. Fifteen of 17 studies showed a statistically significant association between increasing MM and higher mortality. Ten of 14 studies showed no significant associations between MM and HbA1c. Four of 14 studies found higher levels of MM associated with higher HbA1c. Increasing MM was significantly associated with hypoglycaemia in 9/10 studies. There was no significant association between MM and fasting glucose (one study). No studies explored effects on glycaemic variability.

**Conclusions:**

This review demonstrates that MM in T2D is associated with higher mortality and hypoglycaemia, whilst evidence regarding the association with other measures of glycaemic control is mixed. The current single disease focused approach to management of T2D seems inappropriate. Our findings highlight the need for clinical guidelines to support a holistic approach to the complex care needs of those with T2D and MM, accounting for the various conditions that people with T2D may be living with.

**Systematic review registration:**

International Prospective Register of Systematic Reviews CRD42017079500

## Introduction

Type 2 diabetes (T2D) is a leading health priority. Over 424 million people live with diabetes worldwide, leading to $727 billion US dollars in healthcare expenditure [1]. It is estimated that four million people die from diabetes related causes every year, equivalent to one death every eight seconds [1].

T2D management is complex and requires continuous efforts from both clinicians and patients to implement recommendations for self-management and pharmacotherapy to achieve evidence-based targets. For patients who have other chronic conditions in addition to T2D, this complexity is amplified. This is important as T2D rarely occurs on its own–multimorbidity (MM) (presence of ≥ 2 conditions) is the norm, with approximately 85% of those living with T2D having at least one other chronic condition [2].

MM produces many challenges. It is associated with lower quality of life, increased costs, a reduced ability to make lifestyle changes and may be associated with complex therapeutic regimens [3], increasing the treatment burden experienced by the patient [4]. This in turn may challenge and overwhelm individuals, resulting in reduced adherence and poorer outcomes [4]. MM presents health professionals with increased workloads, and the clinical challenge of interactions between multiple conditions and medications (4).

While MM brings about multiple challenges in a clinical sense, it also presents multiple considerations from a conceptual perspective. The terms comorbidity and MM are often used interchangeably [5]. Only more recently has there been a clearer understanding and distinction between the two terms. Comorbidity is defined as the occurrence or existence of an additional condition that co-occurs with an index disease [6]. MM however refers to the presence of two or more chronic conditions in an individual, with no reference to an index condition [7]. The former term illustrates the traditional approach to understanding T2D along with its well-known micro- and macro-vascular complications. The latter term covers a different view where a person’s overall illness burden is the focus and provides the basis for our systematic review, which focuses on MM in people with T2D.

Despite MM presenting many challenges, there is no “gold standard” for the measurement of MM [8]. We still lack clear and comprehensive criteria for the measurement of MM and selection of chronic conditions that qualify for MM. As a result, a numerical count is an acceptable form of measurement of MM, including particular scales (e.g. Charlson Comorbidity Index) [9] that count particular conditions.

MM is common in T2D and brings many challenges, yet currently little is understood about the association between MM and outcomes in T2D. This systematic review seeks to explore literature on the association between MM condition count in people with T2D and the following two primary outcomes: 1) all-cause mortality; and 2) glycaemia (measured by HbA1c). Secondary outcomes of interest include other measures of glycaemia: 1) hypoglycaemia, 2) hyperglycaemia; and 3) glycaemic variability.

## Methods

Our detailed review protocol has been published elsewhere [10] and is summarised below.

We have followed the Preferred Reporting Items for Systematic Reviews and Meta-Analyses Protocols (PRISMA-P) guidelines [11] and our review is registered on the International Prospective Register of Systematic Reviews (CRD42017079500).

### Search strategy

A comprehensive search strategy was used to identify empirical quantitative studies with data on associations between MM condition count and our outcomes of interest in people with T2D. Target studies were observational studies that used either a cross-sectional or longitudinal cohort design. The search was limited to papers published in English but there was no restriction on publication date. A formal database search strategy was developed in consultation with a librarian (PC) from a biomedical library and members of our author group with expertise in MM and T2D, using a combination of free text search and medical subject headings; this is shown in S1 Text. Databases searched were MEDLINE (OVID interface), EMBASE (OVID interface), CINAHL Complete (EBSCO interface), The Cochrane Library (OVID interface), and SCOPUS. The search centred around five key concepts: T2D, MM, comorbidity, glycaemia and mortality. The search was carried out to include literature published up to 28^th^ July 2017.

### Inclusion/exclusion criteria

We included empirical quantitative studies that included data on associations between MM condition count and adults with T2D and all-cause mortality or glycaemic outcomes. Full details of inclusion and exclusion criteria for studies are shown in S1 Table and discussed in detail elsewhere [10].

### Data screening, extraction and analysis

The data screening process was conducted in three stages. Titles of identified studies in the searches were screened by the primary researcher (JC) with a deliberately inclusive approach to reduce the risk of missing potentially relevant studies. Abstract, full paper screening, data extraction and quality appraisal were undertaken by two individual reviewers with any inter-reviewer disagreements resolved by a third reviewer. Covidence [12], a systematic review management software, was used to store, share and assess papers, and undertake selection for inclusion (beyond the title screen). Data were extracted in a structured manner using a predefined data extraction form. Details of the data extraction instrument developed and used are published elsewhere [10]. Data were analysed using a prespecified Population, Exposure, Comparator, Outcomes (PECO) framework which was an adapted framework based on the Cochrane PICO statement where “I” for intervention is replaced with an “E” for exposure. “Study Characteristics” were also included in the framework to take characteristics including study design, setting, period of study, and aims and objectives into account. This PECO approach is acceptable as informed by the Cochrane Handbook [13] and has been utilised previously in a range of published systematic reviews [14, 15]. During data analysis and synthesis, we grouped the included studies according to the two outcomes of interest (all-cause mortality and glycaemia), further subgrouping the latter into different measures of glycaemia. We then considered the implications of the overall findings for each of the outcomes, in order to identify knowledge gaps and clarify the key messages from the evidence, including implications for future research and clinical guidelines for the management of people with T2D.

### Quality appraisal

Quality appraisal was conducted by two reviewers independently. All studies were assessed using the Newcastle-Ottawa quality assessment scale [16] which was informed by recommendations from the Cochrane Handbook on assessing the quality of non-randomised studies [13]. The Newcastle-Ottawa quality assessment scale utilises a star system to judge three broad domains of the included studies: *the selection of the study groups; the comparability of the groups;* and *the ascertainment of the outcome of interest*. We adapted the quality assessment scale to suit our systematic review; shown in S2 Text. The Newcastle-Ottawa quality assessment scale was designed so that it could be modified for specific systematic reviews for non-randomised studies. For example, in the comparability domain, the scale asks the review authors to select the two most important factors that the included studies should control for. The scale’s face/content validity and inter-rater reliability have been established [16] and our approach with the modified scale has been previously used in a published systematic review [14]. Studies were not excluded based on the quality appraisal because our aim was to develop a comprehensive understanding of the existing literature that explored associations between MM and T2D.

## Results

### Retrieved studies

**Fig 1:**
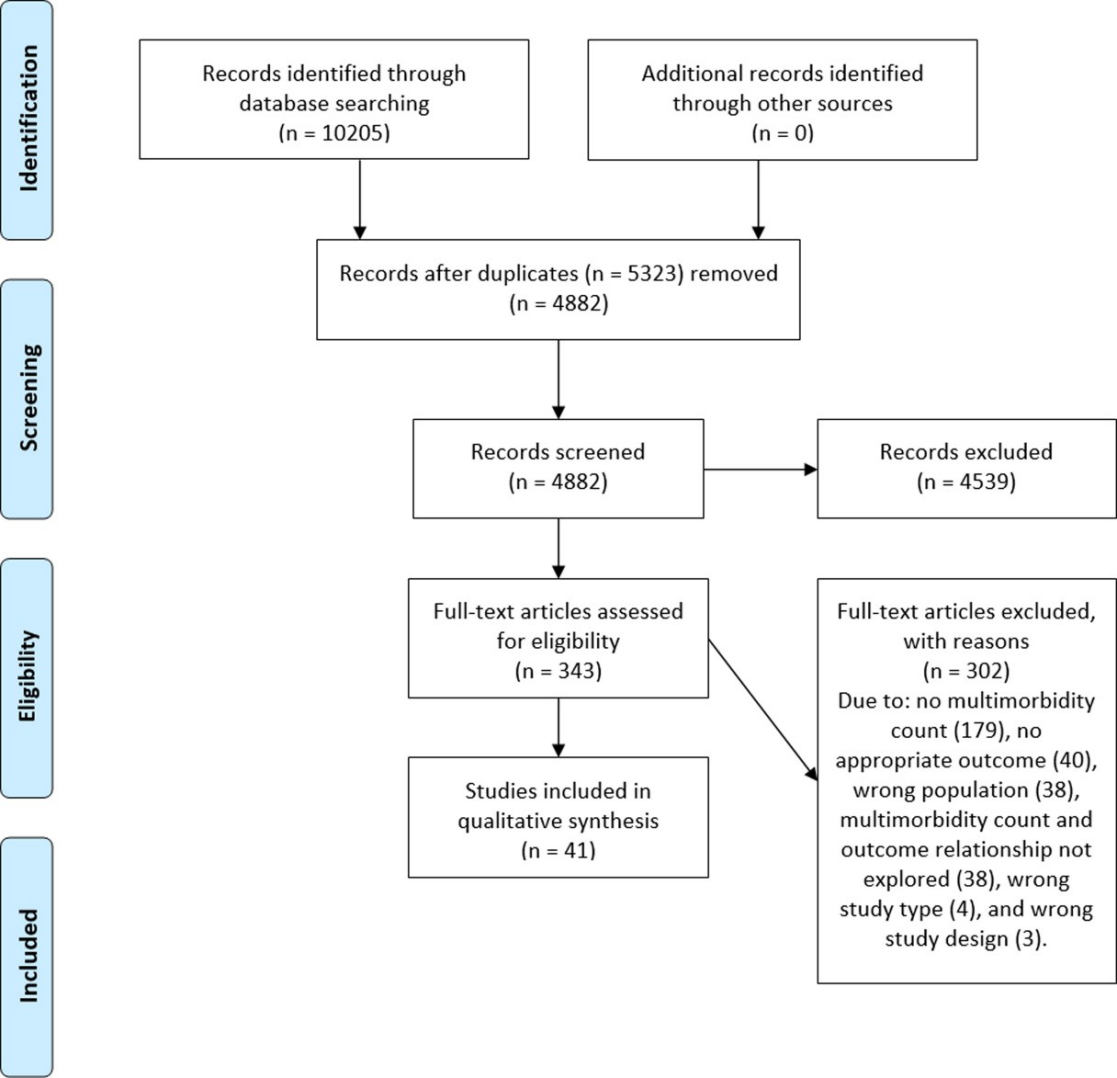
PRISMA flow diagram.

In total, 4,882 papers were identified with our search strategy, and 41 subsequently met our inclusion criteria. Fig 1 demonstrates the inclusion and exclusion of papers at each stage of the screening stages utilising the PRISMA flow diagram [11].

### Included studies and their characteristics

We included 41 studies in our review. Twenty-four studies [17–40] had glycaemia as an outcome, 16 studies [41–56] had all-cause mortality as an outcome and one study [57] included both outcomes.

Key descriptive information of the included studies is summarised in Table 1 below. Participant numbers ranged from 96 to 892,223. There were 28 longitudinal cohort studies [18, 21, 28, 29, 32–37, 40–57] and 13 cross-sectional studies [17, 19, 20, 22–27, 30, 31, 38, 39]. All included studies were conducted in high or upper-middle-income countries.

**Table 1:** Summary of included studies.

Reference	Outcome measure	Country	Study setting and design	Number of participants	MM measure
	**Both**				
Escalada J et al., 2016	All-cause mortality Hypoglycaemic event	US	Community; cohort study	N = 31,035	Charlson comorbidity index
	**All-cause mortality**				
Castro-Rodriguez M et al., 2016	All-cause mortality	Spain	Community; cohort study	N = 363	Charlson comorbidity index
Greenfield S et al., 2009	All-cause mortality	Italy	Diabetes outpatient clinic and primary care; cohort study	N = 2,613	Total illness burden index
Huang YQ et al., 2014	All-cause mortality	China	Hospital; cohort study	N = 533	Charlson comorbidity index
Hunt KJ et al., 2013	All-cause mortality	US	Veterans administrations; cohort study	N = 892,223	Condition count
Kheirbek RE et al., 2013	All-cause mortality	US	Veterans administrations; cohort study	N = 17,773	Severity of illness index
Lin WH et al., 2015	All-cause mortality	Taiwan	Community; cohort study	N = 65,559	Charlson comorbidity index
Lynch CP et al., 2014	All-cause mortality	US	Veterans administrations; cohort study	N = 625,903	Condition count
Martin WG et al., 2014	All-cause mortality	Australia	Hospital; cohort study	N = 210	Condition count
McEwen LN et al., 2012	All-cause mortality	US	Community; cohort study	N = 8,334	Charlson comorbidity index
Monami M et al., 2007	All-cause mortality	Italy	Hospital; cohort study	N = 1,667	Charlson Comorbidity Index and Condition Count
Monami M et al., 2006	All-cause mortality	Italy	Hospital; cohort study	N = 2,002	Charlson comorbidity index
Walker J et al., 2016	All-cause mortality	Scotland	Community; cohort study	N = 126,648	Charlson Comorbidity Index, Elixhauser Comorbidity Index and Condition Count
Wang CP et al., 2014	All-cause mortality	US	Veterans administrations; cohort study	N = 2,415	Charlson comorbidity index
Weir DL et al., 2016	All-cause mortality	US	Community; cohort study	N = 285,231	Condition count
Wilke T et al., 2015	All-cause mortality	Germany	Hospital; cohort study	N = 35,661	Charlson comorbidity index
Zelada H et al., 2016	All-cause mortality	Peru	Hospital; cohort study	N = 499	Condition count
	**Glycaemic outcomes**				
Gallegos-Carrillo K et al., 2009	Fasting plasma glucose	Mexico	Community; cross-sectional	N = 666	Condition count
Abbatecola AM et al., 2015	HbA1c	Italy	Nursing homes; cross-sectional	N = 1,845	Condition count
Bae JP et al., 2016	HbA1c	US	Primary care; cross-sectional	N = 248,567	Charlson comorbidity index
El-Kebbi IM et al., 2001	HbA1c	US	Diabetes clinic; cross-sectional	N = 823	Chronic disease score
Foran E et al., 2015	HbA1c	Ireland	Primary care; cross-sectional	N = 283	Condition count
Fox KM et al., 2006	HbA1c	UK	Primary care; cross-sectional	N = 11,866	Condition count
Frei A et al., 2012	HbA1c	Switzerland	Primary care; cross-sectional	N = 326	Condition count
Hudon C et al., 2008	HbA1c	Canada	Primary care; cross-sectional	N = 96	Cumulative illness rating scale
Luijks H et al., 2015	HbA1c	Netherlands	Primary care; cohort study	N = 610	Condition count
Mosen DM et al., 2017	HbA1c	US	Hospitals and outpatient; cross-sectional	N = 19,600	Charlson comorbidity index
Pollack M et al., 2010	HbA1c	US	Community; cohort study	N = 16,168	Charlson comorbidity index
Romero SP et al., 2013	HbA1c	Spain	Hospital; cohort study	N = 1,519	Charlson comorbidity index
Svensson E et al., 2016	HbA1c	Denmark	Community; cohort study	N = 38,418	Charlson comorbidity index
Teljeur C et al., 2013	HbA1c	Ireland	Primary care; cross-sectional	N = 424	Condition count
Walker RJ et al., 2015	HbA1c	US	Primary care; cross-sectional	N = 615	Charlson comorbidity index
Abbatecola AM et al., 2015	Hypoglycaemic event	Italy	Nursing homes; cohort study	N = 2,258	Condition count
Fonseca V et al., 2017	Hypoglycaemic event	US	Community; cohort study	N = 18,918	Charlson comorbidity index
Kim HM et al., 2016	Hypoglycaemic event	Korea	Community; cross-sectional	N = 307,170	Charlson comorbidity index
Kostev K et al., 2014	Hypoglycaemic event	Germany	Primary care; cohort study	N = 32,545	Charlson comorbidity index
McCoy RG et al., 2013	Hypoglycaemic event	US	Primary care and specialty practices; cross-sectional	N = 326	Charlson comorbidity index
Quilliam BJ et al., 2011	Hypoglycaemic event	US	Hospital; nested case control study	N = 14,729	Charlson comorbidity index
Rathmann W et al., 2013	Hypoglycaemic event	Germany	Primary care; cohort study	N = 50,294	Charlson comorbidity index
Signorovitch JE et al., 2013	Hypoglycaemic event	US	Community; cohort study	N = 33,492	Charlson comorbidity index
Yu HC et al., 2014	Hypoglycaemic event	Taiwan	Hospital; cohort study	N = 399,252	Charlson comorbidity index

### Measure of MM

The measure of MM condition count used in the included studies varied considerably. The majority of studies (24/41 (59%)) [19, 21, 27, 28, 30–37, 39–41, 43, 46, 49–53, 55, 57] measured MM in terms of the Charlson Comorbidity Index (CCI) [9]. MM was represented as a simple count of conditions that were available in the study datasets in 15 studies (32%) [17, 18, 22–25, 29, 38, 44, 47, 48, 50, 52, 54, 56]. The remaining studies measured MM with other indices including the chronic diseases score [20], cumulative illness rating scales [26], total illness burden index [42], severity of illness index [45] and the Elixhauser index [52], or a combination of multiple MM measures [50, 52]. The measure of MM for each of the studies is summarised in S2 Table. This further highlights the heterogeneity of included studies. Meta-analysis was not possible due to variation in measurement of MM.

Only 11/41 (27%) [20, 26, 29, 38, 42, 43, 47, 50, 52, 54, 57] studies had the primary objectives of exploring the relationship between MM and our outcomes of interest, however, all included studies provided data about the association between MM and mortality or glycaemia. 9/41 (22%) studies [24, 27, 28, 33, 37, 39, 46, 49, 56] had broad scoping aims of determining factors that were associated with mortality or glycaemia outcomes. More than half, 21/41 (51%) studies [17–19, 21–23, 25, 30–32, 34–36, 40, 41, 44, 45, 48, 51, 53, 55] did not state the investigation of MM as a research objective, but contained MM count as a covariate when exploring the impact of other factors on mortality or glycaemia, including data allowing us to identify the association between MM count and our outcomes of interest. The research aims included describing the effects of a range of factors (e.g. medication adherence, treatment complexity, anti-diabetic oral treatment, treatment interactions, social support, economic burden, obesity) on glycaemic control and mortality. This wide range of research objectives demonstrates the heterogeneity of the included studies.

Description of the demographics of patients included in the studies is summarised in Table 2 below. The mean age of participants ranged from 38 to 83 years; this was unclear or could not be calculated from the data provided in seven studies [23, 25, 27, 35, 38, 45, 48, 49]. Gender of participants were not described in two studies [23, 49] and in those that did (n = 39) [17–22, 24–48, 50–57], the percentage of female ranged from 2% to 70%. Ethnicity was reported in 11 studies [19, 20, 24, 29, 31, 32, 39, 44, 45, 49, 53] and socioeconomic status was seldom reported (n = 7) [29, 32, 38–40, 52, 54]. Full details of participants are shown in S2 Table.

**Table 2:** Participant demographics.

Reference	Outcome measure	No of participants and gender % F = female M = male	Mean (SD) age in years	Ethnicity	Socioeconomic status
	**Both**				
Escalada J et al., 2016	All-cause mortality Hypoglycaemic event	31035 (53%F 37%M)	72 (9.2)	Not reported	Not reported
	**All-cause mortality**				
Castro-Rodriguez M et al., 2016	All-cause mortality	363 (54.8%F 45.2%M)	76	Not reported	Not reported
Greenfield S et al., 2009	All-cause mortality	2613 (54.8%F 45.2%M)	62.8	Not reported	Not reported
Huang YQ et al., 2014	All-cause mortality	533 (43.4%F 56.6%M)	65.2 (10.8)	Not reported	Not reported
Hunt KJ et al., 2013	All-cause mortality	892223 (2.4%F 97.6%M)	66.2 (11.15)	Non-Hispanic White 61.5%, Non-Hispanic Black 12.1%, Hispanic 13.9%, Other 12.5%	Not reported
Kheirbek RE et al., 2013	All-cause mortality	17773 (4.8%F 95.2%M)	Unclear	White 26.1%, Hispanic 29.9%, Unclear 44%	Not reported
Lin WH et al., 2015	All-cause mortality	65559 (47.9%F 52.1%M)	60.5 (12.9)	Not reported	Not reported
Lynch CP et al., 2014	All-cause mortality	625903 (2.2%F 97.8%M)	65 (11.1)	Non-Hispanic Black 72.1%, Non-Hispanic White 13.2%, Hispanic 5.3%, Other/Unknown race 9.4%	Not reported
Martin WG et al., 2014	All-cause mortality	210 (42.9%F 57.1%M)	Unclear	Not reported	Not reported
McEwen LN et al., 2012	All-cause mortality	8334 (Gender unclear)	Unclear	Non-Hispanic White 50%, Hispanic 15%, African American 18%, Asian/Pacific Islander 9%, Other 8%	Unclear–was included in analysis but not described
Monami M et al., 2007	All-cause mortality	1667 (49.3%F 50.7%M)	65.7 (11.0)	Not reported	Not reported
Monami M et al., 2006	All-cause mortality	2002 (50.1%F 49.9%M)	65.8 (10.8)	Not reported	Not reported
Walker J et al., 2016	All-cause mortality	126648 (44.6%F 55.4%M)	61.9	Not reported	Q1 (most deprived) 23.2%, Q2 22.7%, Q3 20.4, Q4 18.6%, Q5 15.1%
Wang CP et al., 2014	All-cause mortality	2415 (2%F 98%M)	73.7	White/others 83%, Hispanic 7%, Black 10%	Not reported
Weir DL et al., 2016	All-cause mortality	285231 (49.1%F 50.9%M)	53 (10.5)	Not reported	Mean (SD) income in USD $48842 (6567)
Wilke T et al., 2015	All-cause mortality	35661 (54.2%F 45.8%M)	65.91	Not reported	Not reported
Zelada H et al., 2016	All-cause mortality	499 (63.6%F 36.4%M)	61.6 (13.8)	Not reported	Not reported
	**Glycaemic outcomes**				
Gallegos-Carrillo K et al., 2009	Fasting plasma glucose	666 (64.7%F 35.3%M)	Unclear	Not reported	Not reported
Abbatecola AM et al., 2015	HbA1c	1845 (70%F 30%M)	82 (8)	Not reported	Not reported
Bae JP et al., 2016	HbA1c	248567 (50.9%F 49.1%M)	64 (med)	Caucasian 66.5%, African American 14.3%, Asian 2.8%, Other 16.4%	Not reported
El-Kebbi IM et al., 2001	HbA1c	823 (65%F 35%M)	53 (1)	African American 90%, Unclear 10%	Not reported
Foran E et al., 2015	HbA1c	283 (42%F 58%M)	68 (9.5)	Not reported	Not reported
Fox KM et al., 2006	HbA1c	11866 (Gender unclear)	Unclear	Not reported	Not reported
Frei A et al., 2012	HbA1c	326 (42.6%F 57.4%M)	67.1 (10.6)	Swiss 91.8%, Unclear 8.2%	Not reported
Hudon C et al., 2008	HbA1c	96 (51%F 49%M)	66.99	Not reported	Not reported
Luijks H et al., 2015	HbA1c	610 (52%F 48%M)	63 (12.5)	Not reported	Low 52.1%, Middle 40%, High 7.9%
Mosen DM et al., 2017	HbA1c	19600 (48%F 52%M)	63.1	White 78.9%, Hispanic 7.4%, Asian American/Pacific Islander 6.5%, African American 3.9%, Other 3.3%	Not reported
Pollack M et al., 2010	HbA1c	16198 (44.1%F 55.9%M)	52.8	Caucasian 77%, African American 6%, Hispanic 4.3%, Unclear 12.7%	At least 50% had a yearly income >US$65,000 and a net worth of at least US$100,000
Romero SP et al., 2013	HbA1c	1519 (54.2%F 45.8%M)	71.4 (7.6)	Not reported	Not reported
Svensson E et al., 2016	HbA1c	38418 (44%F 56%M)	63	Not reported	Not reported
Teljeur C et al., 2013	HbA1c	424 (46.5%F 53.5%M)	Unclear	Not reported	Low 40.1%, Unclear 59.9%
Walker RJ et al., 2015	HbA1c	615 (38.4%F 61.6%M)	61.3 (10.9)	Non-Hispanic Black 64.9%, Non-Hispanic White 33%, Other/Hispanic 2.1%	Annual income (USD) <$10k 20.2%, $10k-14.9k 11.3%, $15k-19.9k 10.1%, $20k-24.9k 10.4%, $25k-34.9k 14.7%, $35k-49.9k 13.8%, $50k-74.9k 10.1%, $75k+ 9.4%
Abbatecola AM et al., 2015	Hypoglycaemic event	2258 (69%F 31%M)	83 (7)	Not reported	Not reported
Fonseca V et al., 2017	Hypoglycaemic event	18918 (48%F 52%M)	64 (13)	Not reported	Not reported
Kim HM et al., 2016	Hypoglycaemic event	307170 (58.3%F 41.7%M)	Unclear	Not reported	Not reported
Kostev K et al., 2014	Hypoglycaemic event	32545 (49.7%F 50.3%M)	70.2 (11.2)	Not reported	Not reported
McCoy RG et al., 2013	Hypoglycaemic event	326 (44.5F% 55.5%M)	69.3 (12.0)	Not reported	Not reported
Quilliam BJ et al., 2011	Hypoglycaemic event	14729 (46.5%F 53.5%M)	54.8	Not reported	Not reported
Rathmann W et al., 2013	Hypoglycaemic event	50294 (47.1%F 52.9%M)	67.3	Not reported	Not reported
Signorovitch JE et al., 2013	Hypoglycaemic event	33492 (45.3%F 54.7%M)	59.7	Not reported	Not reported
Yu HC et al., 2014	Hypoglycaemic event	399252 (47.4%F 52.6%M)	54.96 (12.51)	Not reported	Monthly salary (NTD), dependants 24.6%, ≤$17280 4.9%, $17281–22800 37.1%, $22801–28800 15.7%, $28801–36300 5.1%, $36301–45800 6.3%, $45801–57800 2.5%, $57801 3.8%

### Quality appraisal

The quality appraisal of the included studies is summarised in S2 Text. Generally, the papers were of moderate to high quality based on the Newcastle-Ottawa quality assessment scale. There were 12 (29%) papers assessed as representative of the general T2D population as they were based on large national datasets. Furthermore, under the *comparability* domain, only 10 (24%) of the 41 included studies considered both age and known duration of diabetes in their data analyses.

### All-cause mortality

We identified 17/41 studies [41–57] that included sufficient data to explore the association between MM condition count and all-cause mortality. All but two studies [41, 52] demonstrated that increasing MM count is associated with statistically significant increased odds ratios (ORs) or hazard ratios (HRs) of death. Studies differed in the analytic methods used to determine the relationship between MM and mortality. These included: two-sample t-test to compare MM between surviving and non-surviving participants; multivariable logistic regression models; and multivariable Cox proportional hazard models. Furthermore, MM was treated in analysis either as a continuous or categorical variable in different studies. For studies that showed a significant increase in mortality whilst treating increasing MM as a continuous variable, the HRs from Cox proportional hazard models that explored MM represented by CCI ranged from 1.22 to 1.95 [50, 51, 53, 55]. Whilst the study that represented MM as total illness burden index, the HR was 1.02 [42]. Studies that reported MM in categories presented more difficulties in comparing the results because they differed in the MM categories that were treated as reference groups [43–49, 54, 56]. This varied from treating 0 conditions, 1 condition, 0–2 CCI and 1–2 CCI as reference in the analyses. The OR results therefore also varied greatly. The OR for mortality was 1.26 for having 1+ conditions in addition to T2D when 0 conditions was the reference group [54], whilst the OR for mortality was 5.46 for CCI of 5+ when CCI of 1–2 was the reference group [43]. The HR results also varied extensively. When CCI of 0–2 was the reference group, the HR for CCI of 3–4 was 1.4 [46]. When having 1 condition in addition to T2D was the reference group, the HR for having 3+ conditions was 21.12 [56]. Despite the heterogeneity of the studies, it is evident that increasing MM count, irrespective of the measure used, is associated with increased all-cause mortality. A summary of the results can be found in S3 Table.

### Glycaemia

We identified 25 studies [17–40, 57] that explored associations between MM condition count and glycaemic outcomes. Fourteen studies [17, 19, 20, 22–24, 26, 29, 31, 32, 35, 37–39] reported HbA1c, 10 studies [18, 21, 27, 28, 30, 33, 34, 36, 40, 57] hypoglycaemia, and one study [25] measured glycaemia in terms of fasting plasma glucose. All results of the included studies are summarised in S3 Table.

#### HbA1c

The majority of studies (10/14)[17, 20, 22–24, 26, 29, 31, 35, 38] showed no association with MM count while four studies [19, 32, 37, 39] found that increased MM count was associated with higher HbA1c. These four studies used different methods of data analysis (S3 Table). Heterogeneity was also seen in the analysis of the effect on HbA1c (continuous for the linear regression model but categorical for the remaining statistical methods).

#### Hypoglycaemia

An increase in MM count was significantly associated with hypoglycaemia in nine of the 10 included studies that presented hypoglycaemia as a glycaemic outcome [18, 21, 27, 28, 33, 34, 36, 40, 57]. Nine of these studies presented MM in terms of the CCI. However, the analysis of data differed greatly (S3 Table). For those that treated MM as a continuous variable using the CCI, the ORs for hypoglycaemia ranged from 1.06 to 1.37 per 1 unit increase in CCI [28, 33]. Similar to the mortality studies, the hypoglycaemia studies that reported MM in categories also presented difficulties in comparing the results due the various reference categories used. One study that used logistic regression models categorised CCI into 1 (reference group), 2, 3, 4 and 5+ with ORs for hypoglycaemia of 1.00, 1.31, 1.81, 2.49 and 4.80, respectively [27]. Another study that used Cox proportional hazard models categorised CCI into 0 (reference group), 1, 2, 3, 4 and 5+; the results showed HRs of 1.00, 1.04, 1.22, 1.16, 1.34 and 1.38, respectively [40]. Despite this heterogeneity, it is evident in the included studies that an increase in MM count is associated with a significantly increased risk of hypoglycaemia.

#### Fasting plasma glucose

One study explored the relationship between MM count and glycaemia as a continuous measure of fasting plasma glucose [25]. The study used a multivariable linear regression model to investigate the association between increasing number of conditions and fasting plasma glucose, and found no statistically significant association.

#### Glycaemic variability

No study reported glycaemic variability as an outcome measure when exploring the relationship between MM count and glycaemia.

### Study heterogeneity

**Fig 2:**
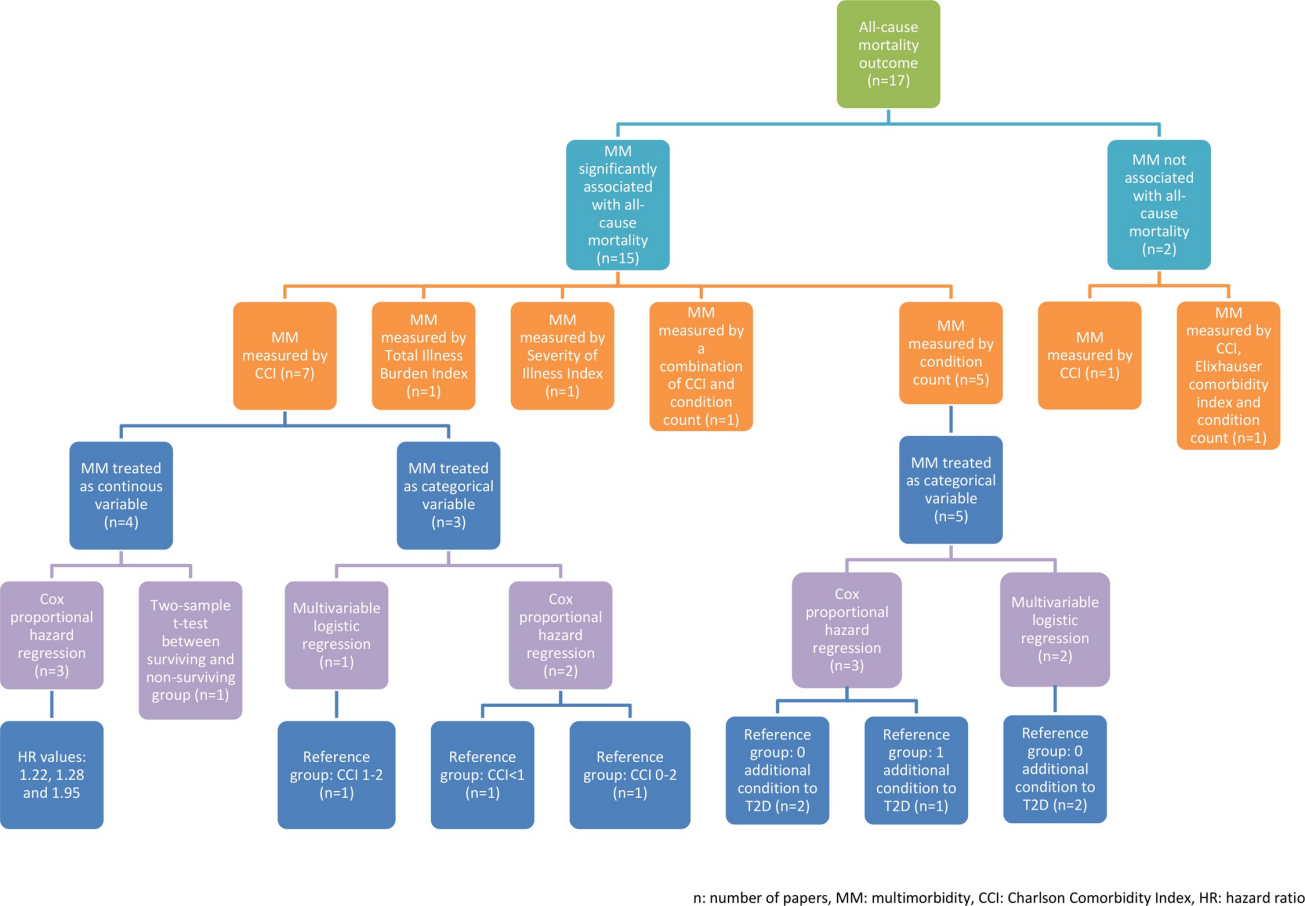
Summary of all-cause mortality outcome papers.

**Fig 3:**
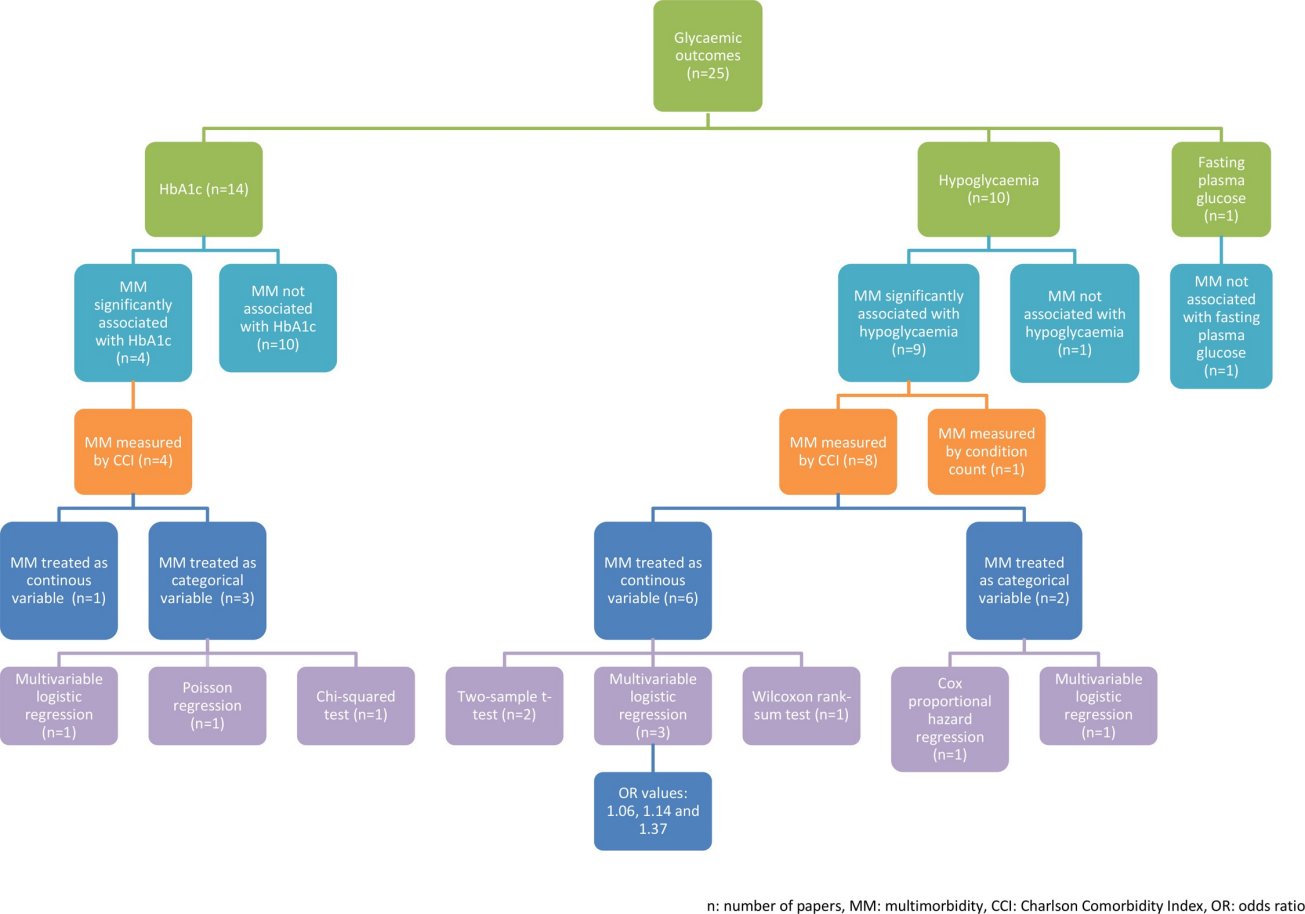
Summary of glycaemic outcomes papers.

Heterogeneity of the included studies can be seen in multiple aspects. It arises from the multiple MM measures used in the included studies, the treatment of MM in data analysis either as continuous or categorical variables and the different statistical analyses used. This is summarised in Figs 2 and 3. As a result, meta-analysis and sub-group analysis are not possible.

## Discussion

### Summary of results

To the best of our knowledge, this is the first systematic review to synthesise the existing evidence on associations between MM, all-cause mortality and glycaemia in people with T2D.

The key findings from our review were that increased MM condition count in those with T2D is significantly associated with all-cause mortality and hypoglycaemia. However, the evidence of effects on other measures of glycaemia including HbA1c and fasting plasma glucose is mixed. No studies explored the associations of MM with glycaemic variability.

### How this fits in with current knowledge

Our findings are consistent with previous literature where increasing MM has been shown to be associated with increased mortality in people with T2D [58–60]. Our findings demonstrated this in a range of settings, countries and study sample sizes. Furthermore, despite variations in the methodologies, measures of MM and statistical analyses, the evidence still suggests an increase in risk of death with increasing MM.

We found no convincing evidence of an association between MM count and HbA1c. This was unexpected as it has been well-established that HbA1c is an important clinical outcome to consider in people with T2D and glycaemic management is a key component of clinical guidelines for T2D. This suggests that the association of MM with mortality that we identified is not necessarily strongly mediated by glycaemia. Studies that have explored the relationship between HbA1c and mortality showed conflicting results where some studies suggested that increased HbA1c was significantly associated with increased mortality [61, 62] however, another demonstrated that the use of intensive therapy to target HbA1c levels below 6.0% increased the rate of death [63]. This mixed picture regarding the relationship between MM count and HbA1c highlights the need for future research to examine how people with T2D and their health professionals approach glycaemic management and targets in the context of MM. It is recognised in many clinical guidelines that HbA1c targets should be individualised based on factors such as age, diabetes duration and MM conditions [64, 65].

We found a significant association between MM and hypoglycaemia with 9/10 studies demonstrating this. Previous studies have suggested that the presence of coexisting conditions may increase a person’s vulnerability to both adverse clinical outcomes, including death, and severe hypoglycaemia [66, 67]. This hypoglycaemia could be as a result of over-treatment or intensive treatment for those that are less healthy (i.e. with greater MM), but a key paper from the Action to Control Cardiovascular Risk in Diabetes (ACCORD) study showed that mortality among those that reported hypoglycaemia was higher for those receiving standard treatment than those receiving intensive treatment [68]. Hypoglycaemia could be a mediating factor for those with increased MM who ultimately have an increased risk of death. It is interesting to note that the one study [30] in our review that reported no association between MM and hypoglycaemia, used self-report hypoglycaemia as an outcome, which was highlighted as a study limitation, as hypoglycaemia awareness may be impaired and / or patient recall may be inaccurate [69, 70].

Another key finding is that despite our comprehensive search of the literature, there was no study that explored the effects of MM count on glycaemic variability. Glycaemic variability, which refers to fluctuations in blood (or interstitial fluid) glucose levels, is a relatively new measure of glycaemia in people with T2D. There is growing interest in targeting reduced glycaemic variability as an independent clinical goal because higher glucose variability is thought to be associated with the development of chronic diabetes complications [71]. It is clear that there is a need for new knowledge to be generated to further understand the association between glycaemic variability and MM, and whether it will be clinically important or just another surrogate marker with little clinical importance to people with T2D.

### Methodological findings and implications

The studies included in our review were highly heterogenous, particularly in the way that MM was measured. There is no consensus as to what is the best method to measure MM, and which is most appropriate to use to predict mortality and other clinical outcomes. Monami *et al*, however, demonstrated that using a condition count was not inferior to the more complex CCI (which applies weightings to the count) when predicting mortality in people with T2D [50]. The attribution of different weights to comorbidities, based on the severity, in the CCI does not seem to add prognostic value in predicting mortality relative to a simple condition count. Moreover, as evident in our findings, increasing counts of conditions and other indices other than the CCI can also be significant predictors of all-cause mortality in people with T2D. Although our findings suggest that various measures of MM showed significant associations with increased mortality, we cannot identify the best measure of MM in predicting mortality. Nor can we identify the type of multimorbid conditions that could have stronger associations with mortality and glycaemia. Piette and Kerr have recommended that multimorbid conditions should be qualitatively assessed as concordant (related to T2D) or discordant (unrelated to T2D) [72] and argue that condition counts are insufficient in describing MM. Therefore, the implications for future research are that well-designed comparative studies of separate measures including concordant and discordant conditions are warranted using large international datasets. Finally, while it will be essential to understand what patterns of MM are associated with the worst outcomes it will also be important to investigate the mechanisms underpinning the increased mortality experienced by those with T2D and MM. Importantly, it will be essential to explore to what extent poor outcomes are explained by biology and how much is a result of health-care systems that may be fragmented and failing to provide coordinated, supportive and holistic care, tailored to the needs of people with complex health care problems [73].

### Strengths and limitations

Key strengths of our review are our adherence to the Preferred Reporting Items for Systematic Reviews and Meta-Analyses Protocols (PRISMA-P) guidelines, our comprehensive search strategy, and that all screening and data extraction were performed by two reviewers independently.

We restricted our search to studies in the English language, which might be viewed as a limitation, although there is increasing evidence that this is unlikely to be a particular problem [74]. We did not apply any geographical limitations, and our review included studies from a variety of countries, all of which were high-income countries or upper-middle-income countries according to the United Nations country classifications. However, there were no studies conducted in low-income countries. We also recognise the limitation of cross-sectional studies in terms of assessment of temporality however, cross-sectional studies provide a snapshot of the relationship between MM count and glycaemia related outcomes of interest.

Important strengths of our review are that we conducted an exhaustive search of five electronic databases and our tight inclusion and exclusion criteria allowed us to gather any data that explored associations between MM and our outcomes of interest, even if the study did not specifically aim to explore this relationship.

A major advantage of our review is that it brings together information about the effects of MM in people with T2D from various sources to create a comprehensive picture of its effects on a number of outcomes. However, one limitation is that the included studies and therefore study participants were highly heterogenous, making comparisons between studies difficult, which prevented meta-analyses and limited us to a narrative synthesis. The large variation arose from the many ways MM is defined, the way outcomes are reported and the varying methods of statistical analyses. Another limitation of our review is that we did not explore the effects of specific multimorbid conditions on our outcomes of interest. While specific comorbidities such as diabetic nephropathy may potentially have contributed to hypoglycaemia as an endpoint in the included studies we were not able to explore this. Our focus is on the overall burden of illness experienced by people with T2D in the form of a MM count. The impact of specific conditions on outcomes in people with MM could be explored in future studies.

## Conclusions

We have reviewed the existing literature to provide a comprehensive summary of the effects of MM count in people with T2D on all-cause mortality and glycaemic outcomes. Our findings show that MM is significantly associated with increased mortality and hypoglycaemia. However, the effects of MM on other measures of glycaemic control, particularly HbA1c, is mixed.

Our review findings emphasise the need for clinical guidelines and clinicians to support a holistic approach to the complex care needs of those with T2D living MM, where care of the whole person should be the primary concern, an approach that accounts for the full range of conditions that people with T2D may be living with.

## Supporting information

S1 Table Inclusion and exclusion criteria for papers. (DOCX)

S2 Table Participant detail. (XLSX)

S3 Table Summary of methods and results. (XLSX)

S1 Text Search strategy. (DOCX)

S2 Text Adapted Newcastle-Ottawa quality assessment scale. (DOCX)

S3 Text PRISMA-P Checklist statement. (DOC)

## Abbreviations

T2D: Type 2 diabetes
MM: Multimorbidity
HbA1c: glycated haemoglobin
GV: Glycaemic variability
CCI: Charlson Comorbidity Index
