# Supplementary material for: Associations between multimorbidity, all-cause mortality and glycaemia in people with type 2 diabetes: A systematic review
Source: PLoS One. 2018 Dec 26;13(12):e0209585. doi: 10.1371/journal.pone.0209585 (PMC6306267; doi:10.1371/journal.pone.0209585)
Supplement: S1 Table — (DOCX) [file pone.0209585.s001.docx]

# Table S1 - Inclusion and exclusion criteria for papers.

*The inclusion and exclusion criteria used during the screening process.*

| **Inclusion Criteria** |  |
| --- | --- |
| Types of studies | No restrictions on publication date. The search end date will be 28 July 2017. |
|  | Studies from any geographical location. |
|  | English language. |
|  | Target studies were studies that use either longitudinal cohort (retrospective and prospective) or cross-sectional designs. |
| Types of participants | Adults (18 years of age or older) with type 2 diabetes (T2D). |
| Types of exposure measures | Multimorbidity (MM) condition count. Any type of MM count, including numerical counts and particular scales. This may include a list of chronic conditions from a variety of datasets including electronic medical records, administrative and prescription datasets. Only studies that assess the relationship between a numerical count of MM and our outcomes of interest were included. |
| Types of outcome measures | All-cause mortality or any glycaemic outcomes. |

| **Exclusion Criteria** |  |
| --- | --- |
| Types of studies | Non English language. |
|  | Grey literature / not published in a peer reviewed journal. |
|  | Dissertations /theses. |
|  | Proceedings. |
|  | Published abstracts. |
|  | Studies using the following methodologies: randomised controlled trials, non-diabetes drug intervention studies, all qualitative studies, case reports and review articles. |
| Types of participants | Children (<18 yrs). |
|  | People without T2D (eg, people with prediabetes, type 1 diabetes/gestational diabetes/monogenic diabetes) |
|  | Animals. |
| Types of exposure measures | Studies with single nominated specific conditions (ie, only one comorbid condition) linked with T2D without MM count. |
