# Supplementary material for: Associations between multimorbidity, all-cause mortality and glycaemia in people with type 2 diabetes: A systematic review
Source: PLoS One. 2018 Dec 26;13(12):e0209585. doi: 10.1371/journal.pone.0209585 (PMC6306267; doi:10.1371/journal.pone.0209585)
Supplement: S1 Text — (DOCX) [file pone.0209585.s004.docx]

# Text S1 – Full Search Strategy

## Full Search Strategy – MEDLINE(OVID) Search Date: 28/07/17

| # | Searches |
| --- | --- |
| 1 | multimorbid* or multi morbid* |
| 2 | condition count* |
| 3 | multiple condition* or multiple disease* or multiple disorder* |
| 4 | multicondition* or multidisease* or multidisorder* or multi condition* or multi disease* or multi disorder* |
| 5 | or/1-4 |
| 6 | diabet* |
| 7 | 5 and 6 |
| 8 | limit 7 to English |
| 9 | animal not human |
| 10 | 8 not 9 |
| 11 | multimorbid* or multi morbid* |
| 12 | condition count* |
| 13 | multiple condition* or multiple disease* or multiple disorder* |
| 14 | multicondition* or multidisease* or multidisorder* or multi condition* or multi disease* or multi disorder* |
| 15 | comorbid* or co morbid* |
| 16 | or/11-15 |
| 17 | glycaem* or glycem* or hyperglycaem* or hyperglycem* or hypoglycaem* or hypoglycem* or glycem* varia* or glycaem* varia* |
| 18 | mortality or death or surviv* or surviv* analys* |
| 19 | (diabetes adj2 ("type 2" or "type ii")) |
| 20 | 19 and 16 |
| 21 | 20 and (17 or 18) |
| 22 | limit 21 to English |
| 23 | 10 or 22 |

## Full Search Strategy – EMBASE(OVID) Search Date: 28/07/17

| # | Searches |
| --- | --- |
| 1 | multimorbid* or multi morbid* |
| 2 | condition count* |
| 3 | multiple condition* or multiple disease* or multiple disorder* |
| 4 | multicondition* or multidisease* or multidisorder* or multi condition* or multi disease* or multi disorder* |
| 5 | or/1-4 |
| 6 | diabet* |
| 7 | 5 and 6 |
| 8 | limit 7 to English |
| 9 | animal not human |
| 10 | 8 not 9 |
| 11 | conference*.pt. |
| 12 | 10 not 11 |
| 13 | multimorbid* or multi morbid* |
| 14 | condition count* |
| 15 | multiple condition* or multiple disease* or multiple disorder* |
| 16 | multicondition* or multidisease* or multidisorder* or multi condition* or multi disease* or multi disorder* |
| 17 | comorbid* or co morbid* |
| 18 | or/13-17 |
| 19 | glycaem* or glycem* or hyperglycaem* or hyperglycem* or hypoglycaem* or hypoglycem* or glycem* varia* or glycaem* varia* |
| 20 | mortality or death or surviv* or surviv* analys* |
| 21 | (diabetes adj2 ("type 2" or "type ii")) |
| 22 | 21 and 18 |
| 23 | 22 and (19 or 20) |
| 24 | limit 23 to English |
| 25 | conference*.pt. |
| 26 | 24 not 25 |
| 27 | 21 or 26 |

## Full Search Strategy – The Cochrane Library (OVID) Search Date: 28/07/17

| # | Searches |
| --- | --- |
| 1 | multimorbid* or multi morbid* |
| 2 | condition count* |
| 3 | multiple condition* or multiple disease* or multiple disorder* |
| 4 | multicondition* or multidisease* or multidisorder* or multi condition* or multi disease* or multi disorder* |
| 5 | or/1-4 |
| 6 | diabet* |
| 7 | 5 and 6 |
| 8 | limit 7 to English |
| 9 | multimorbid* or multi morbid* |
| 10 | condition count* |
| 11 | multiple condition* or multiple disease* or multiple disorder* |
| 12 | multicondition* or multidisease* or multidisorder* or multi condition* or multi disease* or multi disorder* |
| 13 | comorbid* or co morbid* |
| 14 | or/9-13 |
| 15 | glycaem* or glycem* or hyperglycaem* or hyperglycem* or hypoglycaem* or hypoglycem* or glycem* varia* or glycaem* varia* |
| 16 | mortality or death or surviv* or surviv* analys* |
| 17 | (diabetes adj2 ("type 2" or "type ii")) |
| 18 | 17 and 14 |
| 19 | 18 and (15 or 16) |
| 20 | limit 19 to English |
| 21 | 8 or 20 |

## Full Search Strategy – CINAHL Complete (Ebsco) Search Date: 28/07/17

| # | Searches |
| --- | --- |
| 1 | multimorbid* or multi morbid* |
| 2 | condition count* |
| 3 | multiple condition* or multiple disease* or multiple disorder* |
| 4 | multicondition* or multidisease* or multidisorder* or multi condition* or multi disease* or multi disorder* |
| 5 | 1 or 2 or 3 or 4 |
| 6 | diabet* |
| 7 | 5 and 6 |
| 8 | limit 7 to English |
| 9 | multimorbid* or multi morbid* |
| 10 | condition count* |
| 11 | multiple condition* or multiple disease* or multiple disorder* |
| 12 | multicondition* or multidisease* or multidisorder* or multi condition* or multi disease* or multi disorder* |
| 13 | comorbid* or co morbid* |
| 14 | 9 or 10 or 11 or 12 or 13 |
| 15 | glycaem* or glycem* or hyperglycaem* or hyperglycem* or hypoglycaem* or hypoglycem* or glycem* varia* or glycaem* varia* |
| 16 | mortality or death or surviv* or surviv* analys* |
| 17 | diabetes N2 "type 2" |
| 18 | diabetes N2 "type ii" |
| 19 | 17 or 18 |
| 20 | 14 and 19 |
| 21 | 15 or 16 |
| 22 | 21 and 20 |
| 23 | limit 22 to English |
| 24 | 23 or 8 |

## Full Search Strategy – SCOPUS Search Date: 28/07/17

( ( TITLE-ABS-KEY ( diabet* ) ) AND ( TITLE-ABS-KEY ( multimorbid* OR "multi morbid*" OR "condition count*" OR "multiple condition*" OR "multiple disease*" OR "multiple disorder*" OR multicondition* OR multidisease* OR multidisorder* OR "multi condition*" OR "multi disease*" OR "multi disorder*" ) ) OR ( ( TITLE-ABS-KEY ( multimorbid* OR "multi morbid*" OR "condition count*" OR "multiple condition*" OR "multiple disease*" OR "multiple disorder*" OR multicondition* OR multidisease* OR multidisorder* OR "multi condition*" OR "multi disease*" OR "multi disorder*" OR comorbid* OR "co morbid*" ) ) ) AND ( ( TITLE-ABS-KEY ( glycaemia* OR glycemia* OR hypoglycaem* OR hypoglycem* OR hyperglycaem* OR hyperglycem* OR "glycem* varia*" OR "glycaem* varia*" OR mortality OR death OR surviv* OR "surviv* analys*" ) ) ) AND ( ( TITLE-ABS-KEY ( diabetes W/2 "type 2" ) ) OR ( TITLE-ABS-KEY ( diabetes W/2 "type ii" ) ) ) ) AND ( LIMIT-TO ( DOCTYPE , "ar " ) ) AND ( LIMIT-TO ( LANGUAGE , "English " ) )
