# Supplementary material for: Associations between multimorbidity, all-cause mortality and glycaemia in people with type 2 diabetes: A systematic review
Source: PLoS One. 2018 Dec 26;13(12):e0209585. doi: 10.1371/journal.pone.0209585 (PMC6306267; doi:10.1371/journal.pone.0209585)
Supplement: S2 Text — (DOCX) [file pone.0209585.s005.docx]

# Text S2 – Quality Appraisal

| **ID** | **First Author** | **Year** | **Outcome** | **Selection:**  **1)**  **Representativeness of the exposed cohort**  Max 1 | **2)**  **Selection of the non-exposed cohort**  Max 1 | **3)**  **Ascertainment of exposure**  Max 1 | **Comparability:**  **1)**  **Comparability of cohorts on the basis of the design or analysis**  Max 2 | **Outcome:**  **1)**  **Assessment of outcome**  Max 1 | **2)**  **Was follow-up long enough for outcomes to occur (3 years for mortality outcome, 1 year for cohort studies with glycaemic outcomes, n/a for cross sectional studies)**  Max 1 | **3)**  **Adequacy of follow up of cohorts**  Max 1 |
| --- | --- | --- | --- | --- | --- | --- | --- | --- | --- | --- |
| 12 | Escalada | 2016 | Both | * | * | * | * | * |  | * |
| 19 | Gallegos-Carrillo | 2009 | FPG | * | * |  | ** | * | n/a | n/a |
| 1 | Abbatecola | 2015 | HbA1c |  |  | * |  | * | n/a | n/a |
| 5 | Bae | 2016 | HbA1c | * | * | * | ** | * | n/a | n/a |
| 11 | El-Kebbi | 2001 | HbA1c |  |  | * | ** | * | n/a | n/a |
| 15 | Foran | 2015 | HbA1c |  | * |  | * | * | n/a | n/a |
| 16 | Fox | 2006 | HbA1c | * | * | * |  | * | n/a | n/a |
| 17 | Frei | 2012 | HbA1c |  | * |  |  | * | n/a | n/a |
| 24 | Hudon | 2008 | HbA1c |  | * | * | * | * | n/a | n/a |
| 35 | Luijks. | 2015 | HbA1c | * | * | * | ** | * | * |  |
| 46 | Mosen | 2017 | HbA1c | * | * | * | * | * | n/a | n/a |
| 49 | Pollack | 2010 | HbA1c | * | * | * | * | * | * | * |
| 52 | Romero | 2013 | HbA1c |  | * | * |  | * | * | * |
| 54 | Svensson | 2016 | HbA1c | * | * | * | ** | * |  | * |
| 55 | Teljeur | 2013 | HbA1c | * | * | * | * | * | n/a | n/a |
| 57 | Walker | 2015 | HbA1c | * | * | * | ** | * | n/a | n/a |
| 2 | Abbatecola | 2015 | Hypo | * | * | * | ** | * | * | * |
| 14 | Fonseca | 2017 | Hypo | * | * | * |  | * | * | * |
| 28 | Kim | 2016 | Hypo | * | * | * |  | * | n/a | n/a |
| 29 | Kostev | 2014 | Hypo | * | * | * | * | * | * | * |
| 39 | McCoy | 2013 | Hypo | * | * | * |  |  | n/a | n/a |
| 50 | Quilliam | 2011 | Hypo | * | * | * | * | * |  | * |
| 51 | Rathmann | 2013 | Hypo | * | * | * | ** | * | * | * |
| 53 | Signorovitch | 2013 | Hypo | * | * | * |  | * | * | * |
| 61 | Yu | 2014 | Hypo | * | * | * | * | * | * | * |
| 7 | Castro-Rodriguez | 2016 | Mortality | * | * | * | * | * | * | * |
| 20 | Greenfield | 2009 | Mortality | * | * |  | * | * | * | * |
| 23 | Huang | 2014 | Mortality |  | * | * |  |  | * |  |
| 25 | Hunt | 2013 | Mortality |  | * | * | * | * | * | * |
| 27 | Kheirbek | 2013 | Mortality |  | * | * | * | * |  | * |
| 30 | Lin | 2015 | Mortality | * | * | * | * | * | * | * |
| 36 | Lynch | 2014 | Mortality |  | * | * | * | * | * | * |
| 37 | Martin | 2015 | Mortality | * | * | * | * | * |  | * |
| 40 | McEwen | 2012 | Mortality | * | * | * | * | * | * | * |
| 43 | Monami | 2007 | Mortality | * | * | * | * | * | * |  |
| 44 | Monami | 2006 | Mortality | * | * | * | ** | * | * | * |
| 56 | Walker | 2016 | Mortality | * | * | * | * | * |  | * |
| 58 | Wang | 2014 | Mortality |  | * | * | ** | * | * | * |
| 59 | Weir | 2016 | Mortality | * | * | * | * | * | * |  |
| 60 | Wilke | 2015 | Mortality | * | * | * |  | * |  | * |
| 62 | Zelada | 2016 | Mortality |  | * | * | * | * |  | * |

All studies were assessed using the Newcastle-Ottawa quality assessment scale ([1](#_ENREF_1)). We adapted the quality assessment scale to suit our systematic review. We omitted the final item in the selection domain which seeks to determine whether the outcome of interest was or was not present at the start of study. This item was deemed inappropriate for our review where mortality as an outcome would not be present at the start of the study being assessed and the majority of the studies would have glycaemic measures present at the start of the study.

A study can be awarded a maximum of one star for each numbered item within the Selection and Outcome categories. A maximum of two stars can be given for Comparability; one star (*) given if the study controls for age in analysis and two stars (*) given if study controls for both age and duration of diabetes.

1. Wells G, Shea B, O'Connell D, Peterson J, Welch V, Losos M, et al. The Newcastle-Ottawa Scale (NOS) for assessing the quality of nonrandomised studies in meta-analyses [cited 2017 28 July]. Available from: <http://www.ohri.ca/programs/clinical_epidemiology/oxford.asp>.
